# The Importance of Appropriate Dosing of Nonvitamin K Antagonist Oral Anticoagulants for Stroke Prevention in Patients with Atrial Fibrillation

**DOI:** 10.1055/s-0041-1731777

**Published:** 2021-08-23

**Authors:** Jan Beyer-Westendorf, Matthew Fay, Walid Amara

**Affiliations:** 1 Thrombosis Research Unit, Division Hematology, Department of Medicine I, University Hospital “Carl Gustav Carus” Dresden, Dresden, Germany; 2 Department of Haematology, Kings Thrombosis Service, Kings College London, United Kingdom; 3 Westcliffe Medical Practice, Westcliffe Road, Shipley, United Kingdom; 4 Groupe Hospitalier Intercommunal Le Raincy-Montfermeil, Montfermeil, France

**Keywords:** anticoagulants, atrial fibrillation, stroke

## Abstract

Preventing thromboembolic events, while minimizing bleeding risks, remains challenging when managing patients with atrial fibrillation (AF). Several factors contribute to current dosing patterns of nonvitamin K antagonist oral anticoagulants (NOACs), including patient characteristics, comorbidities, and physician judgment. Application of NOAC doses inconsistent with the drug labels may cause patients to receive either subtherapeutic (increasing stroke risk) or supratherapeutic (increasing bleeding risk) anticoagulant levels. In clinical practice, under- or over-dosing of NOACs in patients with AF is not uncommon. This analysis of prospective and retrospective registry and database studies on NOAC use in patients with AF (with at least 250 patients in each treatment arm) showed that under-dosing may be associated with reduced effectiveness for stroke prevention, with similar or even increased bleeding than with the standard dose. This may reflect underlying conditions and patient factors that increase bleeding despite NOAC dose reduction. Such factors could drive the observed overuse of reduced NOAC dosages, often making the prescription of reduced-dose NOAC an intentional label deviation. In contrast, over-dosing more likely occurs accidentally; instead of providing benefits, it may be associated with worse safety outcomes than the standard dose, including increased bleeding risk and higher all-cause mortality rates. This review summarizes the main findings on NOAC doses usually prescribed to patients with AF in clinical practice.

## Introduction

Patients with atrial fibrillation (AF) have a significant, nearly fivefold, increased risk of stroke compared with those without. 1 Oral anticoagulant (OAC) therapy is the cornerstone in prevention of thromboembolic stroke in patients with AF. 2 More than 80% of eligible patients with AF in Europe receive OAC therapy, including vitamin K antagonists (VKAs), such as warfarin, and non-VKA OACs (NOACs), such as apixaban, dabigatran, edoxaban, and rivaroxaban. 3 Both VKAs and NOACs are recommended in clinical guidelines for the prevention of stroke in patients with AF, with a preference for NOACs where suitable. 2

VKAs require frequent monitoring and dose adjustments due to their narrow therapeutic range. 2 4 The broad inter- and intraindividual dose–response variability demands dose optimization of VKA by regular measurements of the prothrombin time, presented as international normalized ratio (INR). The INR target range should be maintained between 2.0 and 3.0, because subtherapeutic INR levels may not provide sufficient protection from stroke. 4 Drug–drug and drug–food interactions and individual metabolic variability may limit the use of VKAs. 4

NOACs have a predictable anticoagulant effect, fixed-dose regimens, and no routine anticoagulation monitoring requirements. 2 The choice and dose of NOAC depend on the individual patient, and appropriate dosing should prevent thrombus formation without increasing major bleeding risk. 5 6 Several factors influence dosing: older age, renal impairment, low body weight, and co-medications, which vary with the NOAC, as indicated in the respective labels. 7 8 9 10

Dose reduction criteria were prespecified in clinical trials. 11 12 13 14 15 16 While NOACs demonstrated a favorable benefit–risk profile in the pivotal phase III randomized controlled trials (RCTs) for stroke prevention in patients with AF, which led to label recommendations for standard dosing and dose reductions, further factors warranting dose reduction may still be identified. In these trials, reductions in the risk of both intracranial hemorrhage and fatal bleeding were observed, compared with warfarin. 11 12 14 15 17 However, real-world studies have shown that administration of anticoagulant therapy is often inconsistent with drug labeling, which may reduce the protective effect of NOACs. 18 19 20 21

Physician preference and experience, patient lifestyle, age, comorbidities, and patient preference contribute to current NOAC dosing patterns. 22 23 Modifying NOAC doses incorrectly can have unintended clinical implications. Suboptimal dosing is associated with poorer clinical outcomes and higher adverse event rates compared with appropriate dosing. 18 19 24 25 Initiating and maintaining the appropriate NOAC dose protects patients against adverse outcomes. 19 24 25 This review summarizes these clinically relevant topics based on prospective and retrospective registry and database studies on the use of NOACs in patients with AF and including at least 250 patients in each treatment arm. Publications were manually screened and additional publications were included according to relevance.

## Criteria for NOAC Dose Adjustments for Patients with Atrial Fibrillation

Advancing age (≥80 years), renal impairment, specific co-medications, and body weight are predisposing factors for increased anticoagulant-related bleeding. 26 A perceived risk of major bleeding can be managed by reducing the dose when risk criteria are present. However, for most patients with AF, stroke risk in the absence of OAC treatment is much higher than bleeding risk associated with treatment. 26

Dose-reduction strategies are important for patients at increased stroke or bleeding risk. Pharmacokinetic analyses from the RE-LY and ENGAGE AF-TIMI 48 trials showed that the probability of major bleeding events increased with increasing NOAC trough plasma concentrations, and the risk of stroke/systemic embolism (SE) was higher at lower plasma trough concentrations. 5 16 Consequently, factors that increase the likelihood of very high or very low NOAC plasma concentrations should be considered when prescribing a NOAC. Patients of older age or with renal dysfunction are at risk of drug accumulation with NOACs. 5 There is limited evidence on the use of NOACs in patients with end-stage renal disease (creatinine clearance [CrCl] < 15 mL/min), and NOACs should be used with caution in patients with severe renal disease when CrCl is 15–29 mL/min (dabigatran is contraindicated for this range). This is because these patients were excluded from the phase III RCTs (ARISTOTLE excluded patients with CrCl < 25 mL/min). 6 27 For moderate renal impairment, a reduced NOAC dose should be considered (please refer to drug- and country-specific label information). 6 This offers a level of protection against stroke, without increasing bleeding risk. If a bleeding event occurs, it may be necessary to interrupt and/or discontinue NOAC treatment, thereby putting the patient at increased risk of potentially fatal thrombosis.

According to the European Union labels, each NOAC has distinct requirements for dose reductions, 26 which may affect treatment decisions ( Table 1 ). 7 8 9 10 28 29 The dose of apixaban may be lowered for patients with serum creatinine level of ≥1.5 mg/dL, which is associated with older age (≥80 years) or low body weight (≤60 kg), and for patients with severe renal impairment (CrCl: 15–29 mg/mL). 7 The dose of dabigatran may be lowered for moderate renal impairment (CrCl: 30–50 mL/min), older age (>75 years and/or high bleeding risk), and concomitant use of P-glycoprotein (P-gp) inhibitors (verapamil, but not amiodarone or quinidine). 8 For edoxaban, the dose may be lowered for moderate to severe renal impairment (CrCl: 15–50 mL/min), low body weight (≤60 kg), and concomitant use of P-gp inhibitors (ciclosporin, dronedarone, erythromycin, or ketoconazole, but not amiodarone, quinidine, or verapamil). 9 Finally, for rivaroxaban dose reductions are required for moderate to severe renal impairment (CrCl: 15–49 mL/min) only, 10 because pharmacokinetic/pharmacodynamic profiles and simulations of drug exposure have shown no effect of age, body weight, gender, or co-medications on drug exposure. 13

**Table 1:** Recommended NOAC dosing for patients with atrial fibrillation 26

	Apixaban a (5 mg bid)	Dabigatran (150 mg bid)	Edoxaban (60 mg od)	Rivaroxaban (20 mg od)
**Age**				
** 75–80 y**	5 mg bid	Consider 110 mg bid	60 mg od	20 mg od
** ≥80 y**	2.5 mg bid with one other factor a	110 mg bid	60 mg od	20 mg od
**Body weight**				
** 50–60 kg**	2.5 mg bid with one other factor a	150 mg bid	30 mg od	20 mg od
** ** **<** **50 kg**	2.5 mg bid with one other factor a	Consider 110 mg bid	30 mg od	20 mg od
**Serum creatinine ≥1.5 mg/dL**	2.5 mg bid with one other factor a	–	–	–
**CrCl**				
** 30–49 mL/min**	5 mg bid	150 or 110 mg bid	30 mg od	15 mg od
** 15–29 mL/min**	2.5 mg bid	Contraindicated/75 mg bid c	30 mg od	15 mg od
** ** **<** **15 mL/min**	Contraindicated	Contraindicated	Contraindicated	Contraindicated
**Concomitant medication** b				
**Cyclosporine**	–	Contraindicated	30 mg od	–
**Dronedarone**	0	Contraindicated/consider 75 mg bid c d	30 mg od	Not recommended
**Erythromycin**	–	–	30 mg od	20 mg od
**Ketoconazole**	Not recommended	Contraindicated/consider 75 mg bid c d	30 mg od	Not recommended
**Verapamil**	5 mg bid	150 or 110 mg bid	60 mg od	–

Abbreviations: bid, twice daily; CrCl, creatinine clearance; NOAC, nonvitamin K antagonist oral anticoagulant; od, once daily.

a For apixaban, 2.5 mg bid is indicated in patients with two or more of the following characteristics: age ≥80 years, body weight ≤60 kg, and serum creatinine ≥1.5 mg/dL (133 µmol/L).

b Incomplete list. See individual labels for more information.

c Dabigatran 75 mg is only available in the United States, and the use of dabigatran in these patients may be contraindicated in other countries.

d In patients with CrCl 30–50 mL/min.

In addition to the inconsistent dose reduction criteria for each NOAC, the extent of dose reductions is also variable. While rivaroxaban and dabigatran are reduced by only approximately 25%, apixaban and edoxaban are reduced by 50%, which may lead to clinically relevant discrepancies of drug exposure and outcomes.

## Evidence for the Use of NOAC Reduced Dose for Stroke Prevention in Patients with Atrial Fibrillation

Evidence for the use of NOACs at the lowest approved dose for stroke prevention in patients with AF has been demonstrated in the four major phase III stroke prevention trials. 27 The proportion of patients with renal impairment who received a reduced dose varied across these trials. 30 31 32 33 Table 2 shows the proportion of patients who received a reduced NOAC dose in these trials, as well as the criteria for dose reduction, and the proportion of patients with moderate renal impairment who received a reduced dose of NOAC.

**Table 2:** Proportion of patients receiving a reduced dose of NOAC across phase III stroke prevention trials

Trial	Normal dose	Proportion of patients receiving reduced dose (dose received)	Criteria for dose reduction	Proportion of patients with moderate renal impairment who received a reduced dose
** ARISTOTLE 14**	5 mg bid	4.7% (2.5 mg bid)	Based on prespecified dose reduction criteria, i.e., ≥2 of: age ≥80 years, body weight ≤60 kg, serum creatinine level of ≥1.5 mg/dL	Substantially lower proportion (24%) of patients with moderate renal function (CrCl ≤50 mL/min) received reduced dose versus other trials 32
** RE-LY 11**	110 mg bid and 150 mg bid (randomization 1/1)	NA	None	Separate datasets for patients with moderate renal impairment based on study design 30
** ENGAGE AF-TIMI 48 12**	Three-arm trial, with 50% of patients receiving 60 mg od and 50% receiving 30 mg od	One-quarter received a reduced dose (30 mg od if randomized to the 60 mg od arm; 15 mg od if randomized to the 30 mg od arm), with ∼75% of these having moderate renal impairment	Half-dose in patients meeting any of the following criteria at screening or at any time during the trial: CrCl 30–50 mL/min, body weight ≤60 kg, or concomitant use of a specific P-gp inhibitor (quinidine, verapamil, dronedarone)	84% of patients with moderate renal impairment (CrCl 30–50 mL/min) at randomization received a reduced dose 31
** ROCKET AF 15**	20 mg bid	21% (15 mg od)	Prespecified dosing criterion: CrCl 30–49 mL/min	Prospective testing allowed for all patients (100%) with a CrCl of 30–49 mL/min to receive the 15 mg od dose 33

Abbreviations: bid, twice daily; CrCl, creatinine clearance; NA, not applicable; NOAC, nonvitamin K antagonist oral anticoagulant; od, once daily; P-gp, P-glycoprotein.

In the phase III stroke prevention trials, patients receiving the lowest doses of dabigatran (110 mg twice daily [bid]) or edoxaban (30 mg once daily [od] or 15 mg od in patients meeting criteria for dose reduction) or reduced doses of apixaban (2.5 mg bid) or rivaroxaban (15 mg od) experienced similar rates of stroke and SE to corresponding patients receiving warfarin. 11 12 14 33 However, in ENGAGE AF-TIMI 48, rates of ischemic stroke were significantly higher in patients on edoxaban 30 mg od compared with those on warfarin. 12 In RE-LY and ENGAGE AF-TIMI 48, patients on the lowest dose of NOAC experienced lower rates of major bleeding compared with those on warfarin. 11 12 In ARISTOTLE and ROCKET AF, the relative safety of reduced-dose NOAC versus warfarin was consistent with the results of the respective trials. 14 33 These findings support the use of the lowest approved doses of NOAC for stroke prevention in a subset of well-defined patients with AF at risk of NOAC overexposure due to various factors. These included low body weight, renal impairment, or concomitant use of certain medications that interact with cytochrome P450 3A4 (CYP3A4) and/or P-gp.

## Prevalence and Clinical Implications of the Use of Nonrecommended Anticoagulant Doses in Clinical Practice

Real-world evidence has shown that many patients receive NOAC doses inconsistent with the label recommendation, including under- and over-dosing (see Table 3 ). 18 Underdosing may lead to inadequate protection from stroke or SE, and a full dose in patients who meet the dose reduction criteria may increase their risk of bleeding. 18

**Table 3:** Prevalence of under- and over-dosing in observational studies

Study	Study type	Patients under-dosed	Clinical impact of under-dosing (HR vs. recommended dose)	Patients over-dosed	Clinical impact of over-dosing (HR vs. recommended dose)
** Yao et al 18**	Retrospective database analysis	13.3% of patients without indication for dose reduction	*Apixaban* Stroke/SE: HR 4.87 (95% CI, 1.30–18.26) Major bleeding: NS *Dabigatran and rivaroxaban* Stroke/SE: NS Major bleeding: NS	43.0% of patients with indication for dose reduction	*Apixaban, dabigatran, and rivaroxaban pooled* Stroke/SE: HR 1.66 (95% CI, 0.40–6.88) Major bleeding: HR 2.19 (95% CI, 1.07–4.46)
** Yu et al 19**	Korea insurance claim analysis	31.2%	*NOACs pooled* Stroke/SE: HR 1.00 (95% CI, 0.91–1.10) Major bleeding: HR 0.99 (95% CI, 0.88–1.11)	8.4%	*NOACs pooled* Stroke/SE: HR 1.16 (95% CI, 1.01–1.34) Major bleeding: HR 1.18 (95% CI, 1.01–1.38)
** García Rodríguez et al 20**	UK electronic health record analysis of patients with NVAF initiating therapy with a NOAC	*Apixaban:* 21.6% *Dabigatran:* 8.7% *Rivaroxaban:* 9.1%	NR	*Apixaban:* 3.5% *Dabigatran:* 16.9% *Rivaroxaban:* 6.6%	NR
** Cho et al 21**	Korea insurance claim analysis	51.6% among newly initiated patients with indication for standard dose	Stroke/SE: 2.38 vs. 2.30% incidence per 1 year (vs. recommended dose) Major bleeding: 1.99 vs. 1.51% incidence per 1 year (vs. recommended dose)	NR	NR
** Steinberg et al 24**	U.S. prospective national registry (ORBIT-AF II)	9.4%	Stroke/SE: 2.0 vs. 1.3 events per 100 patient-years (vs. recommended dosing) Major bleeding: 4.1 vs. 3.6 events per 100 patient-years (vs. recommended dosing)	3.4%	Stroke/SE: 2.3 vs. 1.3 events per 100 patient-years (vs. recommended dosing) Major bleeding: 6.9 vs. 3.6 events per 100 patient-years (vs. recommended dosing)
** Lee et al 25**	Retrospective single center study	19.6%	NR a	6.1%	NR a
** Helmert et al 40**	Prospective registry (Dresden NOAC registry)	64.9% of patients receiving apixaban that had complete information on dosing criteria	NR	7.7% of patients receiving apixaban that had complete information on dosing criteria	NR

Abbreviations: CI, confidence interval; HR, hazard ratio; NR, not reported; NOAC, nonvitamin K antagonist oral anticoagulant; NS, not significant; NVAF, nonvalvular atrial fibrillation; SE, systemic embolism.

a No comparisons between outcomes in appropriate and inappropriate dosing groups were reported, only event rates in appropriate and inappropriate dosing groups vs. warfarin.

### Under-dosing

Discrepancies between the proportion of patients prescribed a reduced NOAC dose in real-world practice compared with patients in phase III RCTs were identified in large NOAC registries and databases 18 and in prescription data analyses. 34 35 Dose reductions occurred in a higher proportion of patients (24.5–53.7%) in real-world studies compared with the phase III stroke prevention RCTs (4.7–25.4%). 27 Differences in under-dosing between the NOACs have also been observed, with one analysis of data from 30,467 patients with nonvalvular AF in UK primary care showing that underdosing was more than twice as likely among patients starting apixaban as in patients starting rivaroxaban or dabigatran. 20

**Fig. 1:**
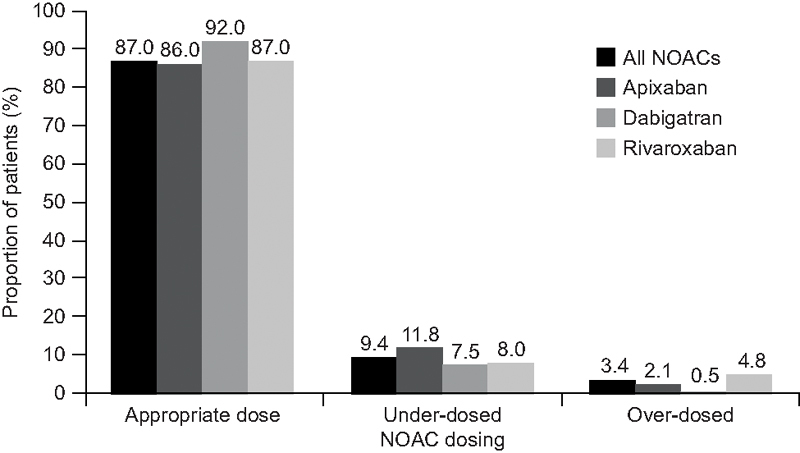
NOAC dosing by drug in the ORBIT-AF II registry. 24 Rates of NOAC prescription, according to the FDA-approved labels with the appropriate dose, below the appropriate dose (under-dosed) and above the appropriate dose (over-dosed). FDA, U.S. Food and Drug Administration; NOAC, nonvitamin K antagonist oral anticoagulant.

The United States-based ORBIT-AF II registry found that 12.9% of patients received nonrecommended NOAC doses according to drug labeling, with 9.4% being under-dosed ( Fig. 1 ). 24 Increased rates of hospitalization for cardiovascular reasons (adjusted hazard ratio [HR] 1.26; 95% confidence interval [CI], 1.07–1.50; *p* = 0.007) were seen in under-dosed patients compared with patients receiving the recommended dose. 24 The highest rates of under-dosing occurred in patients receiving apixaban (12% of the overall population), particularly those on dialysis (29%; according to the U.S. label, patients on dialysis, aged <80 years, and with a body weight >60 kg, can be treated with apixaban 5 mg bid if indicated), and in those with an estimated CrCl of 30–50 mL/min receiving dabigatran (23%). 24

A large U.S. database evaluating NOAC dosing patterns in 14,865 patients with nonvalvular AF found that dosing was often inconsistent with U.S. Food and Drug Administration (FDA) specifications. 18 A total of 13.3% of the 13,392 patients who did not have a renal indication for dose reduction were under-dosed. Of note, outcomes on effective stroke prevention differed between NOACs in this setting. For apixaban-treated patients, under-dosing was associated with an increased risk of stroke/SE (0.54 events per 100 person-years for the standard dose and 2.57 events per 100 person-years for the reduced dose; HR 4.87; 95% CI, 1.30–18.26; *p* = 0.02). 18 In contrast, no significant difference in the risk of stroke or major bleeding was observed for patients without a renal indication for dose reduction treated with reduced doses of dabigatran or rivaroxaban. Bleeding rates remained largely unaffected by dose reduction with all three NOACs. 18 The higher stroke rates observed with apixaban may be the result of the higher number of patients receiving a reduced dose, compared with patients in the rivaroxaban and dabigatran groups; patients receiving apixaban were also older than those receiving the other NOACs (83 years vs. 77 and 76 years, respectively). 18 Additionally, a 50% dose reduction of NOACs in patients with a CrCl >50 mL/min is more likely to result in insufficient drug exposure compared with a 25% dose reduction.

These findings were similar to those from a Danish national cohort study, which showed a trend toward a higher risk of stroke/SE with the reduced dose of apixaban (4.8%), and a trend toward a lower risk of stroke/SE with the lower dose of dabigatran (3.3%) and rivaroxaban (3.5%), compared with warfarin (3.7%) at 1-year follow-up. The rates provided were weighted event rates per 100 person-years. 23 All patients receiving NOACs in this analysis demonstrated no significant risk difference in stroke/thromboembolism risk between the standard and low NOAC doses. 36 These findings matched analyses from two other U.S. databases, which found no significant difference in associated stroke risk between standard and reduced doses of rivaroxaban (20 mg od/15 mg od) versus those of dabigatran (150 mg bid/75 mg bid). 37 38 In the United States, dabigatran 75 mg bid is the approved reduced dose for stroke prevention in patients who have AF and renal impairment (CrCl: 15–30 mL/min and CrCl: 30–50 mL/min if administered with dronedarone or systemic ketoconazole). 39

Several analyses have evaluated the impact of under-dosing in Asian populations. In an analysis from the Korean National Health Insurance Service database, 51.6% of the 16,568 patients with AF who were indicated for a standard dose of a NOAC received a lower dose. 21 Overall, NOAC under-dosing resulted in similar 1-year incidence rates of ischemic stroke or SE compared with standard dosing (2.38 vs. 2.30%, respectively), and increased rates of all-cause death (2.38 vs. 1.59%) and major bleeding (1.99 vs. 1.51%). Although under-dosing with rivaroxaban resulted in a similar risk of thromboembolic events, all-cause death, and major bleeding, the under-dosing of apixaban was associated with an increased risk of ischemic stroke or SE and death. 21 A separate analysis from the same database involving 53,649 patients treated with a NOAC found that under-dosing was not generally associated with either adverse clinical outcomes or improved safety, although under-dosing of rivaroxaban was associated with an increased risk of all-cause death compared with on-label use (adjusted HR 1.37; 95% CI, 1.16–1.63). 19 An analysis from a single center in Korea compared 3,733 patients with nonvalvular AF who were treated with NOACs with 2,659 patients who were administered warfarin. The analysis indicated that NOAC under-dosing was associated with an increased risk of thromboembolism (adjusted HR 2.51; 95% CI, 1.28–4.93). There was no reduction in the risk of major bleeding with under-dosed NOACs compared with the on-label dose in this study. 25

The results for the individual NOACs, however, should not be ignored, and may influence NOAC selection by physicians. For example, the Dresden NOAC Registry found that patients receiving the lower doses of apixaban, rivaroxaban, or dabigatran had more thromboembolic events and significantly more bleeding events compared with those receiving standard doses. 40 41 42 However, these results should be interpreted with caution. Bleeding rates with the reduced doses may be higher because physicians are prescribing these doses to patients most at risk of bleeding. Data from the Dresden NOAC Registry demonstrated that older patients and those with renal impairment or a high HAS-BLED score were more likely to receive rivaroxaban 15 mg od than 20 mg od, indicating a clinical rationale for dose reduction. 42 Therefore, emotionally charged wording such as “inappropriate dosing” should be avoided because, at least in some cases, the prescribed dose may be appropriately driven by clinical considerations, which may have identified bleeding risk factors beyond the label criteria for dose reduction. Another factor in NOAC selection when considering a reduced dose might be the differential proportions of the standard doses used. The reduced doses of apixaban and edoxaban are half the standard dose, compared with 75% of the standard dose for rivaroxaban. For dabigatran, two doses (150 and 110 mg bid) were evaluated in the phase III RE-LY trial, but the lower dabigatran dose (110 mg bid) is only approved in Europe and not in the United States. The United States-approved reduced dose of dabigatran (75 mg bid), which would constitute a similar 50% dose reduction to apixaban, has not been evaluated in any phase III trial of stroke prevention in patients with AF; therefore, physicians may be avoiding prescribing this dose. 18 Physicians may prefer to prescribe apixaban, perceiving it as the lowest dose option in patients at high bleeding risk.

### Over-dosing

In ORBIT-AF II, 3.4% of patients received higher NOAC doses than recommended, especially those with a CrCl of 15–50 mL/min receiving rivaroxaban (34%). 24 Unlike patients who were dosed in accordance with FDA guidelines (87%), nonrecommended NOAC dosing tended to occur more frequently in elderly and female patients, and in those with high CHA _2_ DS _2_ -VASc and ORBIT bleeding scores. 24 Specifically, those over-dosed had both the highest ORBIT bleeding scores and the highest CHA _2_ DS _2_ -VASc scores. 24 Increased all-cause mortality (adjusted HR 1.91; 95% CI, 1.02–3.60; *p* = 0.04) was seen in patients over-dosed compared with those receiving recommended dosing. 24

In the large U.S. database study discussed previously, 43.0% of patients who had a renal indication for NOAC dose reduction ( *n* = 1473) were over-dosed. This was associated with worse safety outcomes and increased bleeding risk (11.29 events per 100 person-years for the standard dose and 5.06 per 100 person-years for the reduced dose; HR 2.19; 95% CI, 1.07–4.46; *p* = 0.03), without a decrease in stroke risk. 18 Taken together, these findings indicate the importance of the correct individual dosing, and that moderate to severe renal impairment (CrCl < 50 mL/min) justifies dose reductions. Additionally, all patients with AF treated with an anticoagulant should have regular CrCl monitoring to detect changes in renal function. 2

In the analysis of the Korean National Health Service database by Yu et al, 8.4% of 53,649 patients with AF treated with a NOAC were found to have been over-dosed. 19 This resulted in an increased risk of stroke or SE compared with standard dosing (HR 1.45; 95% CI, 1.01–1.34), as well as increased risks of major bleeding (HR 1.63; 95% CI, 1.39–1.90) and death (HR 1.81; 95% CI, 1.56–2.09). 19 Conversely, in the single-center study from Lee et al mentioned previously, over-dosing was not associated with any significant difference in either thromboembolism or major bleeding compared with warfarin. 25

## Potential Reasons for the Use of Nonrecommended NOAC Doses in Clinical Practice

### Potential Reasons for Under-dosing

Contributing factors for the current underuse of NOACs, and oral anticoagulation in general, in patients with AF are dependent on the individual patient. Once the physician has accounted for factors such as age, comorbidities, and patient suitability/preferences, the decision may be made not to prescribe oral anticoagulation, and this may be entirely appropriate. 22 Therefore, physicians' judgment and experience in balancing stroke risk against bleeding risk, outside of the CHADS _2_ and CHA _2_ DS _2_ -VASc scores, may explain the discrepancies noted between real-world data and the phase III clinical trials. 23 43 For example, many physicians fear bleeding events and complications, particularly in elderly patients. 20 21 25 26 28 44 45 However, compared with the disabling effects of stroke as a result of withholding anticoagulation, bleeding events are mostly manageable, especially because intracranial hemorrhage is far less common in patients receiving NOACs versus VKAs. Physicians may also be exercising caution in patients close to cutoffs for on-label reduced dose of NOACs on the basis of renal function, body weight, and age. 20 25 Additionally, misperceptions leading to under-dosing of NOACs for safety reasons caused the FDA to cite physician behavior as the primary reason for not approving the lower tested dose of dabigatran (110 mg bid) from the RE-LY trial. 46 Inadvertent under-dosing of the NOACs, which are all P-gp substrates, may arise due to the concomitant use of drugs that induce P-gp expression (such as rifampicin and St John's wort), which has been shown to result in a 35–66% decrease in NOAC plasma levels and, therefore, should be avoided or used with caution. 6

### Potential Reasons for Over-dosing

A reason for NOAC over-dosing could be that physicians are unaware of impaired renal function at the time of prescription or changes to renal function, body weight, age, or co-medication while receiving treatment. Patients' kidney function should be monitored regularly, especially in patients who already have renal impairment, where worsening function is expected, or in the elderly or frail. 2 28 Over-dosing of NOACs may also arise due to concomitant treatment with drugs that increase NOAC plasma levels, such as P-gp inhibitors (all NOACs) and CYP3A4 inhibitors (apixaban and rivaroxaban). Therefore, concomitant treatment with strong inhibitors of both CYP3A4 and P-gp (such as systemic azole-antimycotics [e.g., ketoconazole, itraconazole, and voriconazole] or HIV-protease inhibitors [e.g., ritonavir]) is contraindicated/not recommended in patients receiving apixaban, dabigatran, or rivaroxaban. 6 In patients receiving edoxaban, the 30 mg od dose is recommended with concomitant use of ciclosporin, dronedarone, erythromycin, or ketoconazole (P-gp inhibitors). 9

## Open Questions Relating to NOAC Dosing

Determining the most appropriate OAC (NOAC vs. VKA) and dosing regimens for patients with end-stage renal disease, highly fluctuating renal function, at the extremes of body weight, or with an advanced age is a pressing clinical question. Lack of clinical evidence in these special patient populations requires clinical judgment to determine the most appropriate anticoagulant and dose.

### Changes in Renal Function

The European Society of Cardiology guidelines recommend regular monitoring of renal function during NOAC treatment, to change doses and reassess stroke risk as soon as possible. 2 Deterioration in renal function may require a reduction of the standard NOAC dose to the lower approved dose for stroke prevention, or may even require discontinuation (if CrCl is <30 mL/min).

### Management of Very Obese Patients

The current recommendation for NOACs implies using a fixed dose for obese patients. However, the International Society on Thrombosis and Haemostasis Scientific and Standardization Committee issued a warning against the use of fixed-dose NOACs in patients with a body mass index of > 40 kg/m ^2^ or a weight of > 120 kg, based on lack of evidence for patients at the upper extreme of weight. 47 Subgroup analyses in the large phase III trials suggest that NOACs are efficacious and well tolerated in obese patients; however, the patient numbers were low. 47 The RE-LY trial demonstrated that 1-year stroke/SE rates for patients with AF receiving the standard dabigatran dose (150 mg bid) and the lower dose (110 mg bid) did not differ for the highest body mass index category (>36 kg/m ^2^ ), compared with warfarin. 47 Several case reports of obese patients receiving NOACs experiencing a stroke or SE have suggested that efficacy may be impacted by body weight. 48

Investigation into obese patients with AF and the so-called “obesity paradox” has been performed. 49 The obesity paradox occurs when an inverse relationship is observed between obese patients and a better cardiovascular prognosis. 49 A meta-analysis of the NOAC trials in patients with AF identified lower risks of stroke/SE events and of major bleeding in overweight and obese patients compared with patients of normal weight. 49 This may be partly explained by obese patients being younger than normal-weight patients. In the Dresden NOAC Registry, on-treatment outcomes were lowest in obese patients compared with patients of normal weight, despite obese patients having more cardiovascular risk factors. 50 Conversely, the analysis of a cohort involving 325 patients with AF treated with NOACs indicated that patients with a higher body mass index were more likely to experience both thrombosis and major bleeding earlier than those of normal weight. 51 Recently, a subanalysis of the ENGAGE AF-TIMI 48 trial has been published, alongside several pharmacokinetic/pharmacodynamic studies and large retrospective claims database analyses, which, taken together, provide reassurance that licensed dosing of NOACs is also effective and has a consistent safety profile in very obese patients (body mass index > 40 kg/m ^2^ or body weight > 120 kg). 52 53 54 55 56 57 58 59 It is, therefore, reasonable to expect that the current (2016), more cautious International Society on Thrombosis and Haemostasis guidance will be updated accordingly in the near future.

### Octogenarians and Older Patients

Advancing age is the most common reason for not prescribing anticoagulation in frail patients. 28 Approximately half of all elderly patients with AF have abnormal renal function (52.4%), compared with one-third of elderly patients without AF (32.2%), and this further increases bleeding risk. 44 60 Other comorbidities may also be present, as well as cognitive disorders, risk of falls, and polymedication, all of which can lead physicians to under-dose anticoagulants, 28 despite evidence that the adverse clinical responses associated with under-dosing are also apparent in this population. 45 However, the simple dosing regimens and lower risk of intracranial hemorrhage with NOACs versus VKAs may make them the treatment of choice in the elderly. 28 This is supported by the outcomes of the ELDERCARE-AF trial, which was conducted in Japanese patients ≥80 years of age who were not considered suitable for standard anticoagulation therapy. In this study, low-dose edoxaban significantly reduced the risk of stroke and SE without significantly increasing the risk of major bleeding versus placebo. 61

## Conclusions

Many patients with AF receive NOAC doses inconsistent with the drug label for stroke prevention, which may cause patients to be under- or over-dosed, thereby increasing the risk of adverse clinical outcomes. Use of low-dose NOACs for patients with AF was higher in real-world studies compared with phase III trials. A reduced effectiveness for stroke prevention was observed when low-dose apixaban was used in patients eligible for the standard dose, with no reduction in bleeding risk. Conversely, patients eligible for a low-dose NOAC receiving a standard dose may be at increased bleeding and mortality risk. Anticoagulation in patients with AF can be difficult to manage and regular assessment is required for appropriate dosing and optimal clinical outcomes.

## References

[1] Wolf P A Abbott R D Kannel W B Atrial fibrillation as an independent risk factor for stroke: the Framingham Study Stroke 1991 22 08 983 988 1866765 10.1161/01.str.22.8.983

[2] ESC Scientific Document Group Kirchhof P Benussi S Kotecha D 2016 ESC Guidelines for the management of atrial fibrillation developed in collaboration with EACTS Eur Heart J 2016 37 38 2893 2962 27567408 10.1093/eurheartj/ehw210

[3] Kirchhof P Ammentorp B Darius H Management of atrial fibrillation in seven European countries after the publication of the 2010 ESC Guidelines on atrial fibrillation: primary results of the PREvention oF thromboemolic events--European Registry in Atrial Fibrillation (PREFER in AF) Europace 2014 16 01 6 14 24084680 10.1093/europace/eut263 PMC3864758

[4] Plitt A Bansilal S The nonvitamin K antagonist oral anticoagulants and atrial fibrillation: challenges and considerations J Atr Fibrillation 2017 9 05 1547 29250278 10.4022/jafib.1547 PMC5673394

[5] Ruff C T Giugliano R P Braunwald E Association between edoxaban dose, concentration, anti-Factor Xa activity, and outcomes: an analysis of data from the randomised, double-blind ENGAGE AF-TIMI 48 trial Lancet 2015 385(9984):2288 2295 25769361 10.1016/S0140-6736(14)61943-7

[6] ESC Scientific Document Group Steffel J Verhamme P Potpara T S The 2018 European Heart Rhythm Association Practical Guide on the use of non-vitamin K antagonist oral anticoagulants in patients with atrial fibrillation Eur Heart J 2018 39 16 1330 1393 29562325 10.1093/eurheartj/ehy136

[7] Eliquis ^®^ [Summary of Product Characteristics] Uxbridge: Bristol Myers Squibb, Pfizer Inc; 25 May2021

[8] Pradaxa ^®^ [Summary of Product Characteristics]. Ingelheim am Rhein: Boehringer Ingelheim International GmbH; 22 June 2021

[9] Lixiana ^®^ [Summary of Product Characteristics]. Munich: Daiichi Sankyo Europe GmbH; 23 April 2021

[10] Xarelto ^®^ [Summary of Product Characteristics]. Berlin: Bayer AG; 02 February 2021

[11] RE-LY Steering Committee and Investigators Connolly S J Ezekowitz M D Yusuf S Dabigatran versus warfarin in patients with atrial fibrillation N Engl J Med 2009 361 12 1139 1151 19717844 10.1056/NEJMoa0905561

[12] ENGAGE AF-TIMI 48 Investigators Giugliano R P Ruff C T Braunwald E Edoxaban versus warfarin in patients with atrial fibrillation N Engl J Med 2013 369 22 2093 2104 24251359 10.1056/NEJMoa1310907

[13] Gong I Y Kim R B Importance of pharmacokinetic profile and variability as determinants of dose and response to dabigatran, rivaroxaban, and apixaban Can J Cardiol 2013 29 07 S24 S33 23790595 10.1016/j.cjca.2013.04.002

[14] ARISTOTLE Committees and Investigators Granger C B Alexander J H McMurray J J Apixaban versus warfarin in patients with atrial fibrillation N Engl J Med 2011 365 11 981 992 21870978 10.1056/NEJMoa1107039

[15] ROCKET AF Investigators Patel M R Mahaffey K W Garg J Rivaroxaban versus warfarin in nonvalvular atrial fibrillation N Engl J Med 2011 365 10 883 891 21830957 10.1056/NEJMoa1009638

[16] RE-LY Investigators Reilly P A Lehr T Haertter S The effect of dabigatran plasma concentrations and patient characteristics on the frequency of ischemic stroke and major bleeding in atrial fibrillation patients: the RE-LY Trial (Randomized Evaluation of Long-Term Anticoagulation Therapy) J Am Coll Cardiol 2014 63 04 321 328 24076487 10.1016/j.jacc.2013.07.104

[17] Ruff C T Giugliano R P Braunwald E Comparison of the efficacy and safety of new oral anticoagulants with warfarin in patients with atrial fibrillation: a meta-analysis of randomised trials Lancet 2014 383(9921):955 962 24315724 10.1016/S0140-6736(13)62343-0

[18] Yao X Shah N D Sangaralingham L R Gersh B J Noseworthy P A Non-vitamin K antagonist oral anticoagulant dosing in patients with atrial fibrillation and renal dysfunction J Am Coll Cardiol 2017 69 23 2779 2790 28595692 10.1016/j.jacc.2017.03.600

[19] Yu H T Yang P S Jang E Label adherence of direct oral anticoagulants dosing and clinical outcomes in patients with atrial fibrillation J Am Heart Assoc 2020 9 12 e014177 32495677 10.1161/JAHA.119.014177 PMC7429045

[20] García Rodríguez L A Martín-Pérez M Vora P Appropriateness of initial dose of non-vitamin K antagonist oral anticoagulants in patients with non-valvular atrial fibrillation in the UK BMJ Open 2019 9 09 e031341 10.1136/bmjopen-2019-031341 PMC6756330 31542760

[21] Cho M S Yun J E Park J J Pattern and impact of off-label underdosing of non-vitamin K antagonist oral anticoagulants in patients with atrial fibrillation who are indicated for standard dosing Am J Cardiol 2020 125 09 1332 1338 32098658 10.1016/j.amjcard.2020.01.044

[22] Nabauer M Gerth A Limbourg T The Registry of the German Competence NETwork on Atrial Fibrillation: patient characteristics and initial management Europace 2009 11 04 423 434 19153087 10.1093/europace/eun369 PMC2659602

[23] Nielsen P B Skjøth F Søgaard M Kjældgaard J N Lip G YH Larsen T B Effectiveness and safety of reduced dose non-vitamin K antagonist oral anticoagulants and warfarin in patients with atrial fibrillation: propensity weighted nationwide cohort study BMJ 2017 356 j510 28188243 10.1136/bmj.j510 PMC5421446

[24] ORBIT-AF Investigators and Patients Steinberg B A Shrader P Thomas L Off-label dosing of non-vitamin K antagonist oral anticoagulants and adverse outcomes: the ORBIT-AF II registry J Am Coll Cardiol 2016 68 24 2597 2604 27978942 10.1016/j.jacc.2016.09.966

[25] Lee K N Choi J I Boo K Y Effectiveness and safety of off-label dosing of non-vitamin K antagonist anticoagulant for atrial fibrillation in Asian patients Sci Rep 2020 10 01 1801 32019993 10.1038/s41598-020-58665-5 PMC7000392

[26] Dillinger J G Aleil B Cheggour S Dosing issues with non-vitamin K antagonist oral anticoagulants for the treatment of non-valvular atrial fibrillation: why we should not underdose our patients Arch Cardiovasc Dis 2018 111 02 85 94 28988597 10.1016/j.acvd.2017.04.008

[27] Dobesh P P Fanikos J Reducing the risk of stroke in patients with nonvalvular atrial fibrillation with direct oral anticoagulants. Is one of these not like the others? J Atr Fibrillation 2016 9 02 1481 27909544 10.4022/jafib.1481 PMC5129697

[28] Grupo de trabajo de Riesgo vascular de la SEMI Suárez Fernández C Formiga F Camafort M Antithrombotic treatment in elderly patients with atrial fibrillation: a practical approach BMC Cardiovasc Disord 2015 15 143 26530138 10.1186/s12872-015-0137-7 PMC4632329

[29] Heidbuchel H Berti D Campos M Implementation of non-vitamin K antagonist oral anticoagulants in daily practice: the need for comprehensive education for professionals and patients Thromb J 2015 13 22 26124699 10.1186/s12959-015-0046-0 PMC4484703

[30] Hijazi Z Hohnloser S H Oldgren J Efficacy and safety of dabigatran compared with warfarin in relation to baseline renal function in patients with atrial fibrillation: a RE-LY (Randomized Evaluation of Long-term Anticoagulation Therapy) trial analysis Circulation 2014 129 09 961 970 24323795 10.1161/CIRCULATIONAHA.113.003628

[31] Bohula E A Giugliano R P Ruff C T Impact of renal function on outcomes with edoxaban in the ENGAGE AF-TIMI 48 trial Circulation 2016 134 01 24 36 27358434 10.1161/CIRCULATIONAHA.116.022361

[32] Hohnloser S H Hijazi Z Thomas L Efficacy of apixaban when compared with warfarin in relation to renal function in patients with atrial fibrillation: insights from the ARISTOTLE trial Eur Heart J 2012 33 22 2821 2830 22933567 10.1093/eurheartj/ehs274

[33] Fox K AA Piccini J P Wojdyla D Prevention of stroke and systemic embolism with rivaroxaban compared with warfarin in patients with non-valvular atrial fibrillation and moderate renal impairment Eur Heart J 2011 32 19 2387 2394 21873708 10.1093/eurheartj/ehr342

[34] Gupta M Singh N Tsigoulis M Underuse of full dose Factor Xa inhibition in atrial fibrillation: insight from the SPRINT-AF registry J Am Coll Cardiol 2015 65 A348

[35] Weitz J I Eikelboom J W Appropriate apixaban dosing: prescribers take note JAMA Cardiol 2016 1 06 635 636 27463389 10.1001/jamacardio.2016.1841

[36] Staerk L Gerds T A Lip G YH Standard and reduced doses of dabigatran, rivaroxaban and apixaban for stroke prevention in atrial fibrillation: a nationwide cohort study J Intern Med 2018 283 01 45 55 28861925 10.1111/joim.12683

[37] Graham D J Reichman M E Wernecke M Stroke, bleeding, and mortality risks in elderly Medicare beneficiaries treated with dabigatran or rivaroxaban for nonvalvular atrial fibrillation JAMA Intern Med 2016 176 11 1662 1671 27695821 10.1001/jamainternmed.2016.5954

[38] Hernandez I Zhang Y Comparing stroke and bleeding with rivaroxaban and dabigatran in atrial fibrillation: analysis of the US Medicare part D data Am J Cardiovasc Drugs 2017 17 01 37 47 27637493 10.1007/s40256-016-0189-9 PMC6572759

[39] Pradaxa ^®^ [Prescribing Information]. Ridgefield, CT: Boehringer Ingelheim Pharmaceuticals Inc; July 2020

[40] Helmert S Marten S Mizera H Effectiveness and safety of apixaban therapy in daily-care patients with atrial fibrillation: results from the Dresden NOAC Registry J Thromb Thrombolysis 2017 44 02 169 178 28643004 10.1007/s11239-017-1519-8

[41] Beyer-Westendorf J Ebertz F Förster K Effectiveness and safety of dabigatran therapy in daily-care patients with atrial fibrillation. Results from the Dresden NOAC Registry Thromb Haemost 2015 113 06 1247 1257 25739533 10.1160/TH14-11-0954

[42] Hecker J Marten S Keller L Effectiveness and safety of rivaroxaban therapy in daily-care patients with atrial fibrillation. Results from the Dresden NOAC Registry Thromb Haemost 2016 115 05 939 949 26791999 10.1160/TH15-10-0840

[43] GARFIELD Registry Investigators Kakkar A K Mueller I Bassand J P Risk profiles and antithrombotic treatment of patients newly diagnosed with atrial fibrillation at risk of stroke: perspectives from the international, observational, prospective GARFIELD registry PLoS One 2013 8 05 e63479 23704912 10.1371/journal.pone.0063479 PMC3660389

[44] Ding M Fratiglioni L Johnell K Fastbom J Ljungdahl M Qiu C Atrial fibrillation and use of antithrombotic medications in older people: a population-based study Int J Cardiol 2017 249 173 178 29121723 10.1016/j.ijcard.2017.07.012

[45] de Almeida J PHCL Martinho A S Girão A Novel anticoagulants in an older and frail population with atrial fibrillation: the effect of inappropriate dosing on clinical outcomes Eur Geriatr Med 2020 11 05 813 820 32557249 10.1007/s41999-020-00343-w

[46] Steinberg B A Shrader M A Thomas L Association of inappropriate dosing of non-vitamin K oral anticoagulants and risk of adverse events: results from the ORBIT-AF II registryPaper presented at: European Society of Cardiology congress; Rome, Italy, August 27–31, 2016, Abstract 2954

[47] Martin K Beyer-Westendorf J Davidson B L Huisman M V Sandset P M Moll S Use of the direct oral anticoagulants in obese patients: guidance from the SSC of the ISTH J Thromb Haemost 2016 14 06 1308 1313 27299806 10.1111/jth.13323 PMC4936273

[48] Güler E Babur Güler G Demir G G Hatipoğlu S A review of the fixed dose use of new oral anticoagulants in obese patients: Is it really enough? Anatol J Cardiol 2015 15 12 1020 1029 26663225 10.5152/AnatolJCardiol.2015.6532 PMC5368456

[49] Proietti M Guiducci E Cheli P Lip G YH Is there an obesity paradox for outcomes in atrial fibrillation? A systematic review and meta-analysis of non-vitamin K antagonist oral anticoagulant trials Stroke 2017 48 04 857 866 28265017 10.1161/STROKEAHA.116.015984

[50] Tittl L Endig S Marten S Reitter A Beyer-Westendorf I Beyer-Westendorf J Impact of BMI on clinical outcomes of NOAC therapy in daily care - results of the prospective Dresden NOAC Registry (NCT01588119) Int J Cardiol 2018 262 85 91 29622509 10.1016/j.ijcard.2018.03.060

[51] Lucijanic M Jurin I Jurin H Patients with higher body mass index treated with direct / novel oral anticoagulants (DOAC / NOAC) for atrial fibrillation experience worse clinical outcomes Int J Cardiol 2020 301 90 95 31748190 10.1016/j.ijcard.2019.10.035

[52] Boriani G Ruff C T Kuder J F Relationship between body mass index and outcomes in patients with atrial fibrillation treated with edoxaban or warfarin in the ENGAGE AF-TIMI 48 trial Eur Heart J 2019 40 19 1541 1550 30624719 10.1093/eurheartj/ehy861

[53] Costa O S Beyer-Westendorf J Ashton V Effectiveness and safety of rivaroxaban versus warfarin in obese nonvalvular atrial fibrillation patients: analysis of electronic health record data Curr Med Res Opin 2020 36 07 1081 1088 32347755 10.1080/03007995.2020.1762554

[54] Kushnir M Choi Y Eisenberg R Efficacy and safety of direct oral factor Xa inhibitors compared with warfarin in patients with morbid obesity: a single-centre, retrospective analysis of chart data Lancet Haematol 2019 6 07 e359 e365 31133411 10.1016/S2352-3026(19)30086-9

[55] Martin A C Thomas W Mahir Z Direct oral anticoagulant concentrations in obese and high body weight patients: a cohort study Thromb Haemost 2021 121 02 224 233 32862412 10.1055/s-0040-1715834

[56] Netley J Howard K Wilson W Effects of body mass index on the safety and effectiveness of direct oral anticoagulants: a retrospective review J Thromb Thrombolysis 2019 48 03 359 365 30963393 10.1007/s11239-019-01857-2

[57] Perales I J San Agustin K DeAngelo J Campbell A M Rivaroxaban versus warfarin for stroke prevention and venous thromboembolism treatment in extreme obesity and high body weight Ann Pharmacother 2020 54 04 344 350 31672028 10.1177/1060028019886092

[58] Piran S Traquair H Chan N Bhagirath V Schulman S Peak plasma concentration of direct oral anticoagulants in obese patients weighing over 120 kilograms: a retrospective study Res Pract Thromb Haemost 2018 2 04 684 688 30349887 10.1002/rth2.12146 PMC6178753

[59] Spyropoulos A C Ashton V Chen Y W Wu B Peterson E D Rivaroxaban versus warfarin treatment among morbidly obese patients with venous thromboembolism: comparative effectiveness, safety, and costs Thromb Res 2019 182 159 166 31493618 10.1016/j.thromres.2019.08.021

[60] Nielsen P B Lane D A Rasmussen L H Lip G YH Larsen T B Renal function and non-vitamin K oral anticoagulants in comparison with warfarin on safety and efficacy outcomes in atrial fibrillation patients: a systemic review and meta-regression analysis Clin Res Cardiol 2015 104 05 418 429 25416564 10.1007/s00392-014-0797-9

[61] ELDERCARE-AF Committees and Investigators Okumura K Akao M Yoshida T Low-dose edoxaban in very elderly patients with atrial fibrillation N Engl J Med 2020 383 18 1735 1745 32865374 10.1056/NEJMoa2012883

